# Supplementary material for: A UMLS-based spell checker for natural language processing in vaccine safety
Source: BMC Med Inform Decis Mak. 2007 Feb 12;7:3. doi: 10.1186/1472-6947-7-3 (PMC1805499; doi:10.1186/1472-6947-7-3)
Supplement: Additional file 13 — RAP application directory. Sets up directory for RAP (RDF application for PHP) source code [file 1472-6947-7-3-S13.gz › rap/doc/phpdoc/ontModel/_ontModel_OntModelP_php.html]

xml version="1.0" encoding="iso-8859-1"?


Docs for page OntModelP.php


## /ontModel/OntModelP.php

Description

Description |
Includes

---------------------------------------------------------------------------------- OntModel Package ----------------------------------------------------------------------------------

Description : OntModel package

History: 10-04-2004 : Initial version.

Author: Daniel Westphal <mail at d-westphal dot de>

- filesource: Source Code for this file

Includes

Description |
Includes

include\_once
(RDFAPI\_INCLUDE\_DIR.PACKAGE\_RESMODEL)
(line 17)

require\_once
(RDFAPI\_INCLUDE\_DIR.'ontModel/RdfsVocabulary.php')
(line 19)

require\_once
(RDFAPI\_INCLUDE\_DIR.'ontModel/OntResource.php')
(line 21)

require\_once
(RDFAPI\_INCLUDE\_DIR.'ontModel/OntVocabulary.php')
(line 18)

require\_once
(RDFAPI\_INCLUDE\_DIR.'ontModel/OntProperty.php')
(line 22)

require\_once
(RDFAPI\_INCLUDE\_DIR.'ontModel/OntClass.php')
(line 23)

require\_once
(RDFAPI\_INCLUDE\_DIR.'ontModel/Individual.php')
(line 24)

require\_once
(RDFAPI\_INCLUDE\_DIR.'ontModel/OntModel.php')
(line 20)

Documentation generated on Thu, 7 Jul 2005 13:42:23 +0200 by phpDocumentor 1.3.0RC3
